# Quantum Phase Transition in the Spin Transport Properties of Ferromagnetic Metal-Insulator-Metal Hybrid Materials

**DOI:** 10.3390/nano12111836

**Published:** 2022-05-27

**Authors:** Musa A. M. Hussien, Aniekan Magnus Ukpong

**Affiliations:** 1 Theoretical and Computational Condensed Matter and Materials Physics Group (TCCMMP), School of Chemistry and Physics, University of KwaZulu-Natal, Pietermaritzburg 3201, South Africa; 219042924@stu.ukzn.ac.za; 2 National Institute for Theoretical and Computational Sciences (NITheCS), Pietermaritzburg 3201, South Africa

**Keywords:** 2D materials, magnetoelectric coupling, quantum phase transition, proximity effect

## Abstract

Perpendicular magnetic tunnel junctions provide a technologically important design platform for studying metal-insulator-metal heterostructure materials. Accurate characterization of the sensitivity of their electronic structure to proximity coupling effects based on first-principles calculations is key in the fundamental understanding of their emergent collective properties at macroscopic scales. Here, we use an effective field theory that combines ab initio calculations of the electronic structure within density functional theory with the plane waves calculation of the spin polarised conductance to gain insights into the proximity effect induced magnetoelectric couplings that arise in the transport of spin angular momentum when a monolayer tunnel barrier material is integrated into the magnetic tunnel junction. We find that the spin density of states exhibits a discontinuous change from half-metallic to the metallic character in the presence of monolayer hexagonal boron nitride when the applied electric field reaches a critical amplitude, and this signals a first order transition in the transport phase. This unravels an electric-field induced quantum phase transition in the presence of a monolayer hexagonal boron nitride tunnel barrier quite unlike molybdenum disulphide. The role of the applied electric field in the observed phase transition is understood in terms of the induced spin-flip transition and the charge transfer at the constituent interfaces. The results of this study show that the choice of the tunnel barrier layer material plays a nontrivial role in determining the magnetoelectric couplings during spin tunnelling under external field bias.

## 1. Introduction

Topological matter are exotic quantum systems wherein the notion of topology is required to characterize the collective effects that are observable from their many-body interactions. Their ability to host topological defects and quasiparticles in their lattices is an emergent behavior that makes them unique. This is because the intrinsic character of their broken symmetry states endows them with inherent topological stability against local disorder and external perturbations. The archetypical topological material is the 3D topological insulator whose surfaces and edges act as conductors of carriers due to the presence of chiral edge states while its interior acts as a band insulator due to the presence of a finite excitation gap that is created by the spin-orbit interaction. On the contrary, the closure of the bulk band gap by surface states originates from the nontrivial topology of the bulk electron states. This exotic character originates from an inversion of the topological order in the bulk bands at the time-reversal invariant points of the Brillouin zone denoted by the pair of wave vectors *KK’*. Other examples include topological crystalline insulators, Dirac, and Weyl semimetals. 

Quasiparticle effects on the band gap and the geometric structure of endohedral cagelike clusters were characterized using GW calculations performed within the local density or generalized gradient approximations to density functional theory [1]. This has provided a theoretical basis for understanding the effects of many-body interactions on macroscopic physical properties such as equilibrium geometries, optical polarizabilities, and optical absorption spectra exclusively in terms of the quasiparticle effects on the band gap. Similarly, the band topology and band gap values of the class of 2D materials based on XBi_3_ (X = B, Al, Ga, and In) were shown to depend on both the spin-orbit coupling (SOC) and type of group-III elements in the hetero-sheets [2]. In the XBi_3_ systems, the inclusion of the SOC in the calculations lifts the spin degeneracy of the bands at the Γ point of the Brillouin zone, as would be expected. Crucially, they found that whether the band gap is direct or indirect is also tuned by the SOC and by the type of X element involved. Apart from the intuitive approach of using the topology of bands in the Dirac materials and its sensitivity to the SOC as the bases for understanding their collective effects on the electronic structure when incorporated into artificially stacked ferromagnetic van der Waals systems, we show here that new insights into the transport behavior of the stack are gained by analyzing the asymmetries that characterize the quantum dynamical system of the spin current.

Observing the emergence of a quantum phase transition (QPT) is currently a hot research topic in many-body physics [3]. The QPT occurs at zero-temperature as a distinct change from one ordered quantum state of carriers to another [4,5]. Associated with a QPT in condensed matter systems is the abrupt discontinuity in the local order metric of the Landau theory [6], or the non-local order parameter in the Kosterlitz-Thouless theory [7,8], of phase transitions. Spin-dependent tunneling with perpendicular magnetic tunnel junctions (pMTJs) can be influenced by such abrupt discontinuities due to the introduction of quantum fluctuations into the collective transport of spin from the effects of a finite temperature, an external bias potential, and the applied electric or magnetic field [9]. This is because the design platform and operation of pMTJs permit the simultaneous breakage of structural inversion and time-reversal symmetries. All-spin logic devices (ASLDs) require pure spin currents to function [10,11]. However, the spin polarized current that is generated from pMTJs is highly susceptible to anisotropies, such as those induced by the tunnel barrier material and the applied field. Although several concerted efforts have been focused on the study of different 2D materials as the tunnel barrier layer in pMTJs [12,13,14], neither the nature of the underlying magnetoelectric coupling mechanisms nor the roles of the tunnel barrier layer in the transmitted spin current are well-understood. Gaining insights into such phenomena is a crucial first step toward achieving a lower energy-delay product performance [15]. 

Apart from the dependence of the ASLD-scaling on the engineering of the target interfaces with such tunnel barrier [16], there are several other possible origins of quantum anomalies in the transport of pure spin. These include the spontaneous breaking of the chiral symmetry of carrier spin fields in the presence of electromagnetic fields [17], non-conservation of the rotational symmetry of the spin degree of freedom for carriers at constituent heterobilayer interfaces relative to the spin quantization axis, and the non-conservation of both parity and helicity when spin is projected upon the angular momentum. These broken symmetries are sources of spin anisotropy when the pMTJ design integrates hard ferromagnetic leads with an insulating tunnel barrier layer [15,18], without incorporating a topological insulator to provide the required spin-momentum locking [19,20]. Although insights into the spin field are gained by analyzing the Fermi surface topology for spin carriers [21], field-dependence of the tunneling magnetoresistance (TMR) and its anisotropy [22], and sensitivity of the interfacial spin backflow to tunnel barrier material [23], strategies for mitigating the challenge of energy-delay performance in spintronic devices are still not yet clear. 

Since the isolation of graphene was first reported [24,25], considerable research efforts have been devoted to exploring other forms of 2D materials such as monolayers of group III-V materials and transition metal dichalcogenides due to the high tunability of their electronic structure [26]. The use of hexagonal boron nitride (hBN) [27] or molybdenum disulfide (MoS_2_) [28] as the tunnel barrier region in pMTJs is promising because their wide bandgap creates a high potential barrier between the two potential wells created by the two metallic leads of the reference and free layers. Electrostatic screening of direct interlayer electronic couplings arises when a monolayer tunnel barrier is inserted between the quantum wells created by two fcc Co(111) slabs. Thus, spin carriers must penetrate the barrier to create resonance tunneling as the injected spin-wave density couples to evanescent quantum states [29]. Herein, we investigate emergent carrier transport phenomena in symmetric pMTJs to unravel the effects of the tunnel barrier layer and applied electric field on magnetic proximity couplings in macroscopic spin transport properties under symmetry-breaking fields. This allows for insights into spin anomalies in symmetric pMTJs because such anomalies can introduce resistance through dissipative spin scattering during the operation of ASLDs.

We report a first-order quantum phase transition in the electronic structure of the symmetric Co(111)/monolayer hBN/Co(111) pMTJ system characterized by a sharp discontinuity in the spin transport phase. This results in the change from half-metallic to a metallic character when the magnitude of the applied electric field reaches the critical field of 0.1 Hartree a.u. We gain insights to the electronic phase transition using the spin-flip energy as metric of the local order. Our spin transport calculations show a large tunneling magnetoresistance (TMR) of 139% with monolayer hBN and 987% with monolayer MoS_2_/tunnel barrier. Analysis of the ground state charge transfer shows a contiguous line of zero charge density region surrounding puddles of localized regions of high charged density. The magnitude of the applied electric field at the QPT point corresponds to the electric field at which the TMR saturates in the presence of a monolayer hBN tunnel barrier. We find that the spin current flow in the vicinity of the hBN layer is constrained to a contiguous conducting region of the Co(111)/monolayer hBN interface akin to a spin nanoroad only on one side of the interface due to electric field induced spin crowding. This proximity effect in spin transport occurs through site hopping in the half-metal phase. 

The rest of the paper is organized as follows. In Section 2, we present the theoretical and computational methods used here to calculate the electronic structure in the presence of an applied perpendicular electric field. The results are presented in Section 3 and discussed to unravel the proximity effects in the electronic structure and spin transport properties. In addition, the magnetoelectric coupling from the effect of the applied electric field is also discussed. Lastly, conclusions are presented in Section 4.

## 2. Theoretical and Computational Details

Symmetric pMTJs are modeled here as Co(111)/monolayer tunnel barrier/Co(111) stacks under crystalline epitaxy using approaches that are analogous to those we used recently to study multilayers [18,21,23]. The multilayer stacks studied herein are thermodynamically stable. They denote multilayer stacks that are formed experimentally only through the physical process of the layer-by-layer coupling of free-standing monolayers. The stacks do not form in the gas phase. Instead, they are coupled together by the weak van der Waals forces that acts between the component sheets. This makes the heterostructures to be mechanically stable at 0 K than when compared to forming them in the gas phase. We have performed ab initio electronic structure calculations using the plane wave self-consistent field code version 6.6 (Quantum ESPRESSO Foundation, Italy). This code is a part of the QUANTUM ESPRESSO (QE) package [30,31,32]. Interactions between ions and valence electrons are described using the projector augmented wave method [33]. The exchange-correlation energy was described with scalar relativistic pseudopotentials using nonempirical spin-density van der Waals density (svdw-DF2) functional [34]. Cutoffs of 80 and 450 Ry were set for the kinetic energy and charge density expansions in the plane waves basis. The Brillouin zone was sampled with a 6 × 6 × 1 Monkhorst-Pack k-point grid [35]. However, the much denser mesh of 18 × 18 × 1 k points is used to calculate the projected density of states, with Marzari-Vanderbilt cold smearing width of 0.0074 Ry [36], in each case.

To apply the external electric field, we have added a sawtooth potential to the bare ion Coulomb potential. This approach is different from inserting a dipole layer in the middle of the stack between the vacuum region to simulate a perpendicular electric field [37] or using a gate potential to simulate the effect of an electric field [38]. In each case, the electronic structure is converged self-consistently at the modified potential, but the local structure is not re-optimized at each new value of the electric field. The amplitude of the applied field is given in Hartree atomic units, where 1.0 Hartree a.u. = 51.4220632 × 10^10^ V/m. In each self-consistent calculation of the electronic energy, the total energy was converged to within 10^−6^ Ry and atomic positions were relaxed until Hellman-Feynman forces on each atom are less than 3 meV/Å for all stacks.

Consider that the application of a perpendicular electric field to the pMTJ along the *z*-axis (i.e., parallel to the spin quantization axis) breaks the time reversal symmetry of the ground state spin field. The applied electric field leads to the breaking of the space inversion symmetry of bands in the Brillouin zone. This mechanism is closely associated with the Stark effect on the bands of the Dirac material that is inserted into the ferromagnetic stack. This band inversion is the mechanism that causes a topological phase transition to occur especially at strong field. As shown by Liu et al. [39] in a normal insulator system of few layers of black phosphorus, the above mechanism allows a normal insulator state to be field-tuned into the topological insulator state. This results in spin-separated, and gapless edge states, in the stacked phosphorene structure as would have been expected in the quantum spin Hall effect regime. The applied electric field introduces an extra term to the ground state Hamiltonian, *H*_0_. The expectation value of *H*_0_ is the total energy obtained from the DFT when no electric field is applied while the extra term is the Rashba spin orbit Hamiltonian [40,41].

(1) $$\;H_{R}=\frac{\lambda_{R}}{2}\left(p\times \hat{z}\right)\cdot \sigma$$

Parameters σ and $\lambda_{R}$ denote the Pauli spin matrices and amplitude of the SOC, respectively. Note that $\lambda_{R}$ depends on the amplitude of the applied electric field and the crystal structure of the integrated materials. The resulting ***k***-space Hamiltonian,$\;H=H_{0}+H_{k}$, where $\;H_{k}=\frac{\lambda_{R}}{2}\left(k\times \hat{z}\right)\cdot \sigma$ describes a group of Bloch electrons that move adiabatically as a wavepacket in a nondegenerate band of index *n* of total energy *E*_n_. We treat the wavepacket to include a range of quantized wavevectors that are much smaller than the size of the Brillouin zone. This implies that the dimension of the wavepacket is far larger than the lattice constant in real space. 

To keep the effects of the applied field on the Berry phase tractable in our calculations, we have followed the formalism of Ref. [42]. We have supposed that the wavepackets are localized within the Brillouin zone compared to the amplitude of the applied field such that the position of the electron can be associated with the wavevector k, then the equation of motion under an applied electric (**E**) and magnetic (**B**) fields, in a semiclassical approximation, is given by

(2) $$\frac{dr}{dt}=\left(\frac{2\pi }{h}\right)\frac{\partial E_{n}\left(k\right)}{\partial k}-\frac{dk}{dt}\times \Omega_{n}\left(k\right)$$

where $\Omega_{n}$ is a pseudovector in k-space denoted by the Berry curvature. Note that **B** = 0 in this work. The applied electric field $E\;=\hat{z}E$ modifies the dynamics of carriers significantly and generate new magnetoelectric properties in the transport phenomena by setting up a net magnetic field in momentum space, known herein as the Berry curvature, $\Omega_{n}\left(k\right)$. Since the magnetic field is the curl of the vector potential in position space, the Berry curvature can be written as the curl of the momentum space vector potential,$\;\Omega_{n}\left(k\right)=\nabla_{k}\times A\left(k\right)$, which is denoted by the corresponding Berry connection, A(k)=⟨un,k|i∇kun,k⟩=−Im⟨un,k|∇kun,k⟩. Integrating the Berry connection over the entire Brillouin zone yields the Berry phase, $\Phi \;=\oint A_{n}\left(k\right)\cdot dk$ [43].

The resulting acceleration of spin carriers in the wavepacket associated with the change in the momentum due to the Lorentz force is,

(3) $$F=-e\left[E-\frac{1}{2c}\left(\frac{dr}{dt}\times \left(\nabla \times i\langle u_{n,k}|\nabla_{k}|u_{n,k}\rangle \right)+\nabla \times i\langle u_{n,k}|\nabla_{k}|u_{n,k}\rangle \times \frac{dr}{dt}\right)\right]$$

The Berry curvature associated with the acceleration is

(4) $$\Omega_{n}\left(k\right)=i\frac{h^{2}}{4\pi^{2}m^{2}}\overset{occupied}{\underset{i\neq n}{\sum }}\frac{P_{n,i}\left(k\right)\times P_{i,n}\left(k\right)}{{\left(\left[E_{n}^{0}\left(k\right)-E_{i}^{0}\left(k\right)\right]\right)}^{2}},\;$$

where $P_{n,i}\left(k\right)=u_{n,k}|\hat{p}|u_{n,k}$ denotes the expectation value of the interband matrix element of the momentum operator $\hat{p}$ in the eigenstate $|\psi_{n,k}\rangle =e^{-ik\cdot r}|u_{n,k}\rangle ,\;$where $|u_{n,k}\rangle$ is the periodic part of the Bloch function. Incorporation of hBN and MoS_2_ guarantees the presence of broken inversion symmetry, which allows the existence of an orbital magnetic moment, $m_{n}$. Note that $E_{n}^{0}\left(k\right)$ represents the dispersion of the nth band. It is related to the total energy $E_{n}\left(k\right)$ of carriers as

(5) $$E_{n}\left(k\right)=E_{n}^{0}\left(k\right)-m_{n}\left(k\right)\cdot B$$

where $m_{n}\left(k\right)$ denotes the orbital magnetic moment,

(6) $$\;m_{n}\left(k\right)=-i\frac{eh}{4\pi m^{2}}\overset{occupied}{\underset{i\neq n}{\sum }}\frac{P_{n,i}\left(k\right)\times P_{i,n}\left(k\right)}{\left[E_{n}^{0}\left(k\right)-E_{i}^{0}\left(k\right)\right]}$$

Because of the broken structural inversion symmetry, the effects of the applied electric field are determined by the set of parameters $\Omega_{n}\left(k\right)\;$and $m_{n}\left(k\right),\;$which characterize the effect of the changes in the Berry phase of electrons in the Bloch bands on carrier transport properties. The above effective field theory is used herein to develop a fundamental understanding of the spin dynamics and the magnetoelectric properties in heterostructure multilayers from the proximity effect of the coupling of the electric field to the Berry phase of Bloch electrons. 

Quantum transport properties were calculated using an effective field theory that combines density functional theory with the plane waves basis set for expanding electron wave functions. This approach has been implemented in the PWCOND code of the QE package [44,45] for the study of quantum systems with open boundary conditions containing semi-infinite left and right electrodes with the scattering region at the center [46,47]. Nevertheless, the same carrier transport problem is also solvable self-consistently using localised basis sets within the field-theoretic framework by implementing the non-equilibrium Green’s function formalism. This computational strategy is equivalent to the non-relativistic limit of the quantum electrodynamical density functional QEDFT [48,49,50,51]. As recently implemented in GPAW, an electromagnetic environment can be embedded into state-of-the-art electronic structure methods efficiently through the radiation-reaction potential [52]. This allows for calculation of radiative emission (lifetimes, photoabsorption cross-section, superradiant linewith, etc) from real-time time-dependent density functional theory (TDDFT) using the basis set formed by linear combination of atomic orbitals.

The dense 60 × 60 × 1 k-mesh was used to calculate the wavevector-dependent spin transmission spectrum. The spin-resolved conductance is obtained using the Landauer-Büttiker formalism [53,54]:

(7) $$\;G_{\sigma }=\frac{e^{2}}{h}\underset{k_{∥}}{\sum }T_{\sigma }\left(k_{∥},E_{F}\right),\;$$

where$\;G_{\sigma }$ denotes the conductance per spin channel, $T_{\sigma }\left(k_{∥},E_{F}\right)\;$denotes the transmission coefficient and the summation is over the two-dimensional Brillouin zone denoted by Bloch wave vector $k_{∥}=$ ($k_{x},k_{y}).$ In addition, $E_{F}\;$denotes the Fermi energy, while *e* and *h* denote the electron charge and the Planck’s constant, respectively. The spin-resolved transmission coefficient at $E_{F}$ is calculated using:

(8) $$T_{\sigma }\left(E_{F}\right)=\;T_{r}\left[\Gamma_{L,\sigma }\left(E_{F}\right)G_{\sigma }^{r}\left(E_{F}\right)\Gamma_{R,\sigma }\left(E_{F}\right)G_{\sigma }^{a}\left(E_{F}\right)\right],\;$$

where $G_{\sigma }^{r}$ and $G_{\sigma }^{a}$ are the retarded and advanced Green’s functions, respectively. The linewidth parameter $\Gamma_{\alpha }\;\left(\alpha =L,\;R\right)$ gives the coupling strength between each lead and the scattering region. 

To model the transmission of spin carriers in pMTJs, we assume that the spin degree of freedom is conserved in the tunneling process so that the TMR ratio is $\mathrm{TMR}\;=\frac{G_{P}-G_{AP}}{G_{\mathrm{AP}}}\times 100\%\;$[9], where $G_{P\;}\;$and $G_{\mathrm{AP}}$ denotes the conductance for the parallel (P) and anti-parallel (AP) configurations of the electrodes, respectively. Here, $G_{\;}=\frac{e^{2}}{h}(T^{↑}+T^{↓}$), where $T^{↑}$ and $T^{↓}$ denotes majority-spin and minority-spin conductance, which is obtained in the parallel or anti-parallel configurations from the transmission spectrum at the Fermi level, where $\frac{e^{2}}{h}$ is the conductance quantum. This framework allows us to also analyze the proximity effect due to TMR. Magnetoelectric couplings from proximity effects were also calculated using the ground-state electron density. Interactions between the tunnel barrier and the Co(111) leads were determined in terms of the interfacial charge distribution. The charge transfer (Δρ) due to the covalent bonding and the magnetoelectric couplings due to the proximity effect of the electric field strength was determined as $\Delta \rho \left(r\right)=\rho_{\mathrm{Het}}\left(r\right)-\rho_{\mathrm{Co}}\left(r\right)-\rho_{X}\left(r\right)$. In this case, $\rho_{\mathrm{Het}}\left(r\right)$ denotes the electron density for the multilayer and $\rho_{\mathrm{Co}}\left(r\right)$ denotes the charge density when the tunnel barrier atoms have been removed with all the Co atoms frozen in their positions as in the heterostructure. The charge density $\rho_{X}\left(r\right)$ is calculated for monolayer tunnel barriers when all Co atoms have been deleted from the supercell when all atomic positions in species X = MoS_2_ (or hBN) are frozen. 

## 3. Results and Discussion

### 3.1. Breaking the Ground-State Symmetry

#### 3.1.1. Local Network Structure in Multilayer Heterostructures 

**Figure 1:**
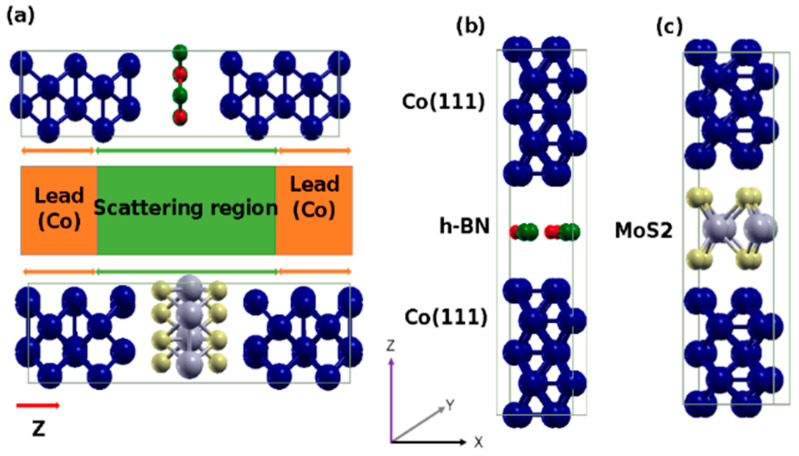
The schematic of the multilayer heterostructures with leads and scattering region, where vector **Z** denotes the axis of spin quantization and the spin transport direction (**a**). The unit cell showing the optimized local structure in the Co(111)/hBN/Co(111) stack, where B and N atoms are shown in green and red (**b**), and the optimized local structure in the Co(111)/MoS_2_/Co(111) stack, where Mo and S atoms are shown in grey and yellow (**c**).

Figure 1 shows the schematic of the multilayer heterostructures (Figure 1a) and the cubical unit cell of the relaxed configuration in Co(111)/hBN/Co(111) (Figure 1b) and Co(111)/MoS_2_/Co(111) (Figure 1c) heterostructure. The central region or tunnel barrier region (shown in green color) is made of a monolayer of either the 2H-polytype of molybdenum disulfide (MoS_2_) or hexagonal boron nitride (hBN). Figure 1a denotes the schematic of a typical device in measurement configuration. In both heterostructures, the right and left leads (shown in orange color in Figure 1a) are each made from 3 atomic layers of the (111) surface of fcc Co. Our results show that the interlayer distance is *d*_Co(111)/hBN_ = 3.38 Å between the Co(111) and hBN layers (see Figure 1b). The distance between the Co(111) and hBN layers are the same for the top and bottom leads and these agree well with the results of other calculations [19,24,55,56]. However, the distance between Co(111) and the upper layer S atoms of the MoS_2_ layer is *d*_Co(111)/MoS2_ = 2.28 Å (see Figure 1c). This also agrees well with other calculations [57,58,59,60], confirming that the ground state electronic structure of the artificial multilayers is properly set up. 

The pMTJs investigated herein are both symmetric because both the right (R) and left (L) leads are each made of Co(111) slabs of 5 atomic layers (inset of Figure 1a). The magnetization in the Co(111) slab of the R-lead is fixed to its value in bulk Co. As such, this layer is denoted as the reference layer. By contrast, magnetization of the Co(111) slab of the L-lead is allowed to reorient itself freely as it responds to the changes in the angular momentum of the spin-polarized electrons that are injected into it after tunnelling through the monolayer tunnel region. This Co(111) layer is denoted as the free layer. The only difference between the two pMTJ models is that the monolayer tunnel barrier region is either hBN or MoS_2_. 

We have also studied the proximity effect of the tunnel barrier layer in terms of changes in the local structure by studying the variations in the Co-Co distances in the reference and free layers. We find that the bond lengths in the ferromagnetic fcc phase of Co(111) remain unchanged. Here the interatomic distances are equal in both metallic leads of the symmetric pMTJ. The length of the metallic Co-Co bonds is unchanged because of the invariance between the fixed and free layers. Consider that Matar, et al. [61] show that at equilibrium the ground state in metallic cobalt is ferromagnetic and that the hcp phase was more stable than the fcc phase. However, the calculated nearest-neighbor distances are found here to be slightly lower than the 2.54 Å distance expected in the most stable but nonmagnetic fcc phase.

At the limit of vanishingly small (or zero) spin orbit coupling (SOC), spin-up and spin-down carriers are unaware that time reversal symmetry has been broken in the pMTJs. Thus, an external bias voltage is the only agency that transmits information about the broken time reversal symmetry to the orbital degree of freedom, and this causes orbital currents to develop, leading to the Berry phase polarization. In a 2D electron gas system, this phenomenon manifests as the anomalous Hall conductivity. In the pMTJ, the integration of a ferromagnet and 2D materials means that time reversal symmetry must be broken spontaneously in a way that allows a component of magnetization to appear in a direction normal to the plane to obtain the quantum anomalous Hall state. Breaking of time reversal symmetry implies an emergent phenomenon wherein a small field-induced change in the ground-state electronic density causes quantum fluctuations in the spin current.

The motion of spin carriers in an electric field is time-reversal invariant but not in a magnetic field. As we will demonstrate in the next section, our calculations show that the zero- and finite-field ground states of the pMTJs are both magnetically ordered. This implies the presence of spontaneous broken time-reversal symmetry in the spin space. The effect is that the finite-field electronic system finds that it is favorable to lower its total energy relative to a fully spin-paired nonmagnetic state. Typically, this is attained by developing unequal populations of spin-up and spin-down electrons on some of the atomic sites. This is observed herein as ‘localized’ magnetic moments on specific atoms, as expected in band magnetism even though bulk Co exhibits itinerant magnetism. The magnetic moments develop a well-defined spin order as expected in ferromagnetism or antiferromagnetism, to minimize the energy of exchange interactions between the two collinear spin configurations. We have determined the magnetoelectric couplings and spin ordered transport state by considering field-induced changes in the parallel and anti-parallel spin configurations and the resulting proximity coupling effects.

Recently, similar nonlinear responses in charge conductivity have been explored by the inclusion of an electric field in the Hamiltonian of a 2D system [62]. In this case, the time-reversal symmetry is also broken by internal mechanisms. Crucially, their model predicts a quantized bulk conductivity with strong dependence on the intensity of the field. In the pMTJs, the underlying quantum fluctuations make the dynamics of spin carriers to cross a boundary from one carrier transport phase to another when a potential bias is introduced. This phase crossing determines whether anisotropies occur in the spin transmission spectrum and decides whether the transport state is either half-metallic or metallic. In the Anderson theory of symmetry-breaking, this phase boundary point is equivalent to the critical point of the continuous quantum phase transition (CQPT). The CQPT is driven exclusively by quantum fluctuations at zero-temperature. The above framework captures the changes in the transport properties that arise when a spin current is flowing and the accompanying magnetic proximity couplings effect. In Section 3.3.2, we demonstrate the sensitivity of these changes to the tunnel barrier layer material and show that the changes reflect anisotropies in the spin current. 

#### 3.1.2. Localized Magnetic Moments

**Figure 2:**
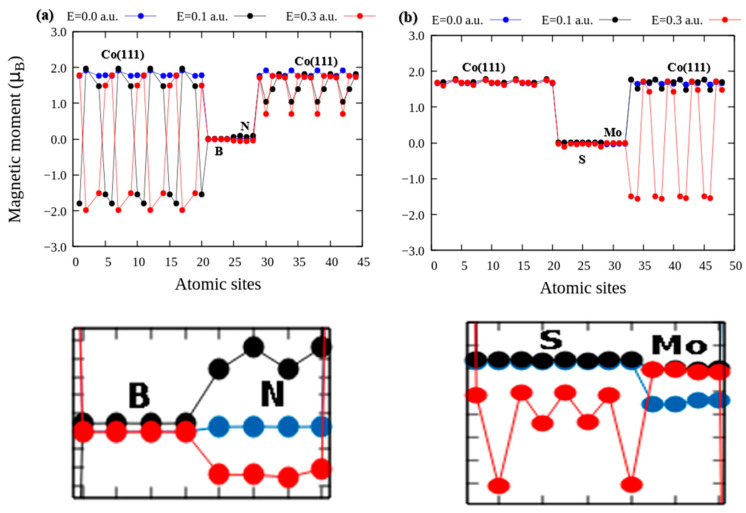
Magnetic spin moments in (μB) per atomic sites (**a**) Co(111)/hBN/Co(111), and (**b**) Co(111)/MoS_2_/Co(111) interfaces. The different colors (blue, black, and red) show the values of the electric field in (a.u.) as a function of the magnetic moment. The green double arrow under and above refers to the atoms at specific positions. The corresponding lower panels represent the zoomed-in image of the central region of the magnetic tunnel junction to show distributions of localized magnetic moments in monolayer hBN and MoS_2_, respectively.

Figure 2 show the distribution of local magnetic moments (*m*) for different amplitudes of the perpendicular electric field (*E*). The ground state local spin moments (shown in blue circles in Figure 2) show minimal variations around the same average irrespective of the nature of the tunnel barrier layer. The local relaxation of the site-resolved magnetic moments reveals a strong sensitivity to the tunnel barrier layer. The local magnetic moment at the ground state is used here as a descriptor of the electronic structure to capture the effect of broken time-reversal symmetry. This is because it exhibits large changes within the reference layer with changing electric field in the presence of monolayer hBN but shows no effect with monolayer MoS_2_. In the free layer, the applied field induces variations in local magnetic moments systematically, and the magnitude of the changes captures the explicit breaking of the symmetry of the ground state electronic structure.

Figure 2a shows that applied electric fields of amplitude 0.1 and 0.3 a.u. cause the local magnetic moments of the bottom Co(111) layers to oscillate between –1.80 and –1.97$\;\mu_{B}$. These oscillations resemble the spatial variations in moments observed in the antiferromagnetic ordering with opposite spin directions. In contrast, the variations in the spin magnetic moments of the top atomic layers in the Co(111) fixed layer are from 0.71 to 1.81 $\mu_{B}$. The Co spin moments, though aligned parallel to the spin quantization axis as would be expected in ferromagnetic moments, exhibit strong layer-dependent oscillations in the presence of the hBN tunnel barrier. However, the local spin moments are 0.005 $\mu_{B}$ at the N atoms. This is increased to 0.092 $\mu_{B}$ at 0.1 a.u. This then reduces to −0.051 $\mu_{B}$ at higher field *E* = 0.3 a.u. (see the bottom panel of Figure 2a). We attribute the observed changes to the electronic couplings induced by the applied perpendicular electric field [13]. In the presence of strong magnetization from the Co(111) layers, the inter-layer electronic couplings culminate in interfacial charge transfer between the Co(111) and hBN layers, and these lead to a slight polarization of the nonmagnetic B, N, Mo, and S atoms.

Figure 2b shows the local spin moments of the Co(111)/MoS_2_/Co(111) stack. Our results show that the bottom Co layers are nearly unchanged under field variation. Fluctuations in the localized magnetic moments occur mainly in the top atomic layers of the free-layer Co, especially at 0.3 Hartree a.u. At zero bias field, the S and Mo atoms magnetic moments are 0.006 and −0.031 $\mu_{B}$, respectively (see bottom panel of Figure 2b). These increase to 0.0083 $\mu_{B}$ for S atoms at 0.1 a.u. parallel to the magnetic moments localized on Mo species. At a higher electric field of 0.3 a.u., comparatively large opposite spin moments are induced on the Mo and S atoms as shown in the bottom panel of Figure 2b. The magnetic moments localized on Mo atoms are negligible. This is attributable to the weak interaction between Mo and Co atoms. In addition, the S atoms of the MoS_2_ tunnel barrier region become slightly polarized while the Mo atoms remain unpolarized. Generally, our results shed light on how magnetic moments are affected by an introduced electric field especially in the tunnel barrier regions which becomes marginally polarized because of magnetic coupling due to the proximity effect induced close to the interface by the Co atoms of the Co(111)/tunnel barrier heterobilayer. 

### 3.2. Quantum Phase Transition 

#### 3.2.1. Non-Volatile 180° Reversal of Magnetization 

Hereunder, we show that a full reversal of magnetization is accompanied by a spin-flip transition in the transport phase. We obtain insights into the origin of the localized magnetic moments induced on constituent atoms of the non-magnetic tunnel barrier region, as indicated in the lower panels of Figure 2. To proceed, we first analyse the response of the local structure to the applied electric field and study the evolving magnetic order. Secondly, we analyse the changes in the local density of states (DOS) for the majority and minority spin electrons due to changes in the strength of the applied electric field. We identify the sudden change in the electronic structure as a first-order quantum phase transition (QPT). This is crucial because the flowing spin current breaks time-reversal symmetry, whereas the application of the external electric field does not. Deeper insights into the electric field-induced changes in the magnetically ordered ground state are obtained by evaluating magnetic ordering energy as Δ*E* = *E*(AP) − *E*(P), where *E*(AP) and *E*(P) are the total energy of the ground state in the parallel (P) and anti-parallel (AP) configurations, respectively. We thus adopt the Heisenberg Hamiltonian $H=-2J\overset{N}{\underset{i,j}{\sum }}S_{i}S_{j}$ to interpret the field-induced band magnetism, where *J* is the magnetic exchange coupling. Here *S*_i_ denotes the total spin magnetic moment at atomic site *i* obtained as the vector sum over all the spins of unpaired electrons, with *i* and *j* labeling two nearest-neighbor sites [63]. 

Table 1 shows the effect of electric field on the magnetic ordering of the ground state of the multilayer heterostructure in terms of the ground state total energy for magnetization in P and AP configurations. The magnetic ordering energy decreases as the electric field is increased when monolayer hBN is inserted in the scattering region. The total magnetization of the P (or AP) configuration simultaneously decreases (or increases) nonlinearly with an increase in the amplitude of the applied field for both tunnel barrier materials. In the MoS_2_ system, the magnetization also shows similar nonlinearities in both P and AP configurations. The value of Δ*E* drops below zero at 0.1 a.u., and this unique point corresponds to the critical value at which the applied electric field simultaneously induces a first-order QPT and a saturation in the TMR in the hBN barrier model. We show in Section 3.2.2 that the electronic structure is characterized by a QPT from a half-metal to full-metal phase in Co(111)/hBN/Co(111) at the critical electric field. With monolayer MoS_2_ as the tunnel barrier, Δ*E* exhibits an opposing trend as with hBN. It drops (rises) rapidly from zero to −2.59 eV (0.038 eV) at the same amplitude of the field of 0.1 a.u. before increasing to −1.95 eV at 0.3 a.u.

Table 1 also shows that the decreasing total magnetization observed in the P-configuration with monolayer hBN becomes even more pronounced in the AP-configuration as a change in the sign of the magnetization vector. These effects on the tunnel barrier are stronger in MoS_2_ than hBN. The observed negative total magnetization denotes a reversal of magnetization through proximity effects. This is a unique signature of the spin-flip transition necessary to realize a nonvolatile 180° magnetization reversal in the spin-transport phase from magnetoelectric and interlayer couplings. Several recent experiments indicate that magnetic moments are induced in non-magnetic elements by the proximity effect of interlayer and magnetoelectric coupling. This electric field-controlled non-volatile reversal of magnetization is traditionally observed in ferromagnetic multiferroic heterostructures [64,65,66], and in antiferromagnets under both collinear [67] and non-collinear [68] magnetizations. The reverse process wherein changes in the direction of magnetization produce spin current through the transfer of angular momentum constitutes spin pumping [69]. The observed dependence of both Δ*E* and *M* on the applied electric field is consistent with the results of our previous calculations of the dynamical response of the Fe/hBN-based tunnel junctions induced by applied axial fields wherein the two metallic leads are made of dissimilar ferromagnets [19].

Table 2 shows the sensitivity of the spin order and magnetic exchange coupling to the tunnel barrier material and the applied electric field. In this case, the magnetization energy (Δ*E*) is mapped on the Heisenberg model for magnetic ordering, $\Delta E=2J{\left|S\right|}^{2},$ where *S* denotes the total spin. The spin order of the transport phase is not sensitive to MoS_2_, but it changes from half-metallic to metallic transport phase at applied field of 0.1 a.u. when the hBN is used. Similarly, the magnetic exchange interaction *J* shows non-linear dependence on electric field and the insulating barrier type. With hBN (MoS_2_) barrier, the magnitude decreases with applied field without a change in sign. Its sign change at the signals also is also a unique signature of the underlying magnetic coupling. 

Cobalt atoms exhibit itinerant magnetism since the unpaired electrons of the 3*d* band state are delocalized. This makes it appear unphysical to use the ‘localized’ magnetic moments model for band magnetism. We now justify the validity of using the Heisenberg model of ferromagnetism to explain band magnetism. Insofar as the ground-state electron density that yields the “effective field” in the calculations is obtained self-consistently amidst the expected charge sloshing in DFT calculations of multilayer heterostructures, it is the equality between the net magnetization in both models that constitutes the appropriate descriptor of the magnetic state of the carrier transport system. For the Heisenberg model, establishing the one-to-one correspondence is important for describing materials whose magnetism is close to the itinerant regime. The need to keep magnetic moments as the same in both models guarantees that any suitable convergence of the magnetic ground state guarantees *a posteriori* validity of our mapping of the itinerant magnetism onto the magnetic exchange coupling parameter of the Heisenberg model. The above interpretation is physically plausible since the charge sloshing effects that arises from the lack of balance between long- and short-ranged charge re-distribution are rectified self-consistently during the iterative update of the electronic charge density.

#### 3.2.2. Magnetoelectric Coupling

##### Field Effects on Spin Dynamics

The case of spin tunneling through a thin insulating tunnel barrier in pMTJs is analogous to the regular flow of carriers in a normal metal in the direction parallel to an external electric field. Nevertheless, time-reversal symmetry is not broken when charge carriers flow through a normal metal. In such scenarios, an applied external electric field exerts a force on the free charge carriers causing a net motion of carriers through the conducting medium as an electric current. The heat energy generated during the flow of current is due to resistance to the rapid propagation of electric energy by an energy-carrying field in the metal. Recently, we used the external magnetic field to break the time-reversal symmetry due to the strong SOC by modeling the spin Hall effect in related heterostructures that contain Pt and Pd. By mimicking the longitudinal flow of the spin current, the spin texture of the carrier transport phase is clarified as a magnetic skyrmion [70]. 

Evidence of the broken time-reversal symmetry in this process is observable in the energy dissipated as heat. Thus, the interplay among the charge, spin, and the dissipated heat are central to the related mechanisms of anomalous Nernst and spin Seebeck effects in spin Hall devices, through which spin-polarized current or pure spin current can be generated and detected. So far, our results indicate that the applied electric field induces tunable magnetic moments within the nonmagnetic hBN layer. The bottom panel of Figure 2a shows variations in the average magnetic moments of the monolayer hBN from 0 (0 a.u.), +0.08 (0.1 a.u) and to −0.06 (0.3 a.u.). The average variations between positive and negative magnetic moments typify the field-induced toggling of magnetic moments relative to the ground state. Thus, rather than using a current or magnetic field for the switching, a full 180° reversal of magnetization is achieved using an applied external electric field. In this approach, the interlayer magnetoelectric couplings in the device do not require a multiferroic material. This approach also provides an affirmative answer to the following fundamental question: can an applied external electric field induce nonzero magnetization in a non-magnetic material.

Our analysis shows that although the electronic structure of the hBN layer is intrinsically nonmagnetic at zero fields, its responses to the magnetic proximity coupling effect of the external field play nontrivial roles in the spin-flip transition. This assertion agrees with recent observations that the interfacial layer dominates the spin-dependent charge transfer in multilayer heterostructure devices [71]. Also, the magnetic moment from the proximity exchange field is typically sensitive to the layered magnetic structure of the whole composite heterostructure. Spin-dependent tunneling in pMTJs is strongly influenced by the bonding at the ferromagnet/insulator interface, Coulomb blockade, magnetic field potential barrier, and applied voltage, amongst other factors [9]. Thus, it is equally relevant to gain insights into the dependence of the electronic properties on electric fields to understand the origin of the QPT. To unravel magnetoelectric coupling in the correlated ground state and the QCP, we have investigated the interplay between the hybridization of orbitals, the applied electric field, and the spin transport by analyzing the projections of the electronic DOS on orbitals of specific atoms. 

##### Projected Density of States

**Figure 3:**
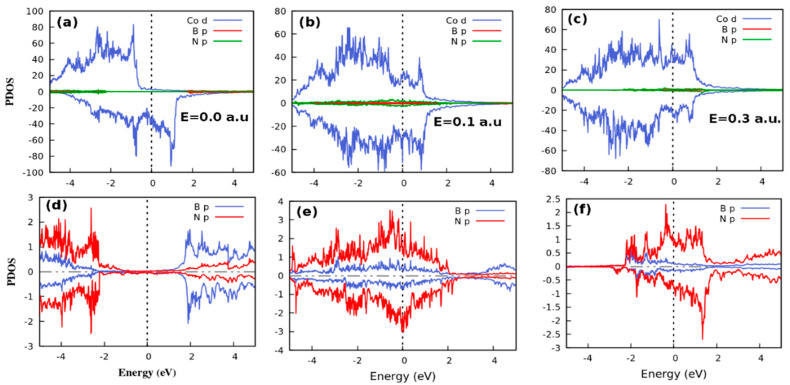
Effect of applied electric field on the spin-polarized orbital-resolved electronic DOS in the monolayer hBN based pMTJ at electric of 0.0 (**a**), 0.1 (**b**) and 0.3 a.u. (**c**), respectively (top panels). The PDOS within the monolayer hBN tunnel region is also displayed in (**d**–**f**) to show the *p*-orbitals of B and N atoms (bottom panel). Positive and negative PDOS denote spin-up and spin-down channels, respectively. The vertical dashed lines indicate the Fermi level (*E_F_*).

**Figure 4:**
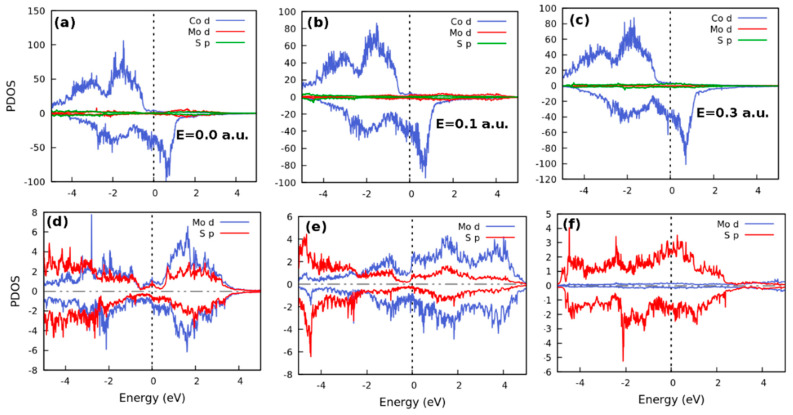
Effect of applied electric field on the spin-polarized orbital-resolved electronic DOS of Co/MoS_2_/Co stack at electric field of 0.0 (**a**), 0.1 (**b**) and 0.3 a.u. (**c**), respectively. The PDOS within the monolayer MoS_2_ tunnel barrier region is also displayed in (**d**–**f**) to show the Mo *d*-orbital and S *p*-orbital at the three different magnitudes of the applied electric field. Positive and negative PDOS denote spin-up and spin-down channels, respectively. The vertical dashed lines indicate the Fermi level (*E_F_*). The vertical dashed lines indicate the Fermi level (*E_F_*).

Figure 3 and Figure 4 show the PDOS of stacks containing hBN and MoS_2_ respectively. Both plots show the PDOS for spin up (positive DOS) and spin down (negative DOS) as calculated when the spins are constrained in a parallel magnetic configuration under the influence of an electric field. The top panels in Figure 3 show the effect of applied external electric field on the PDOS of the Co(111)/hBN/Co(111) multilayer while the hBN layer PDOS is shown in the bottom panels. Figure 3a shows that there are ~40 states per eV/unit cell in the spin-down channel and a vanishing DOS in the spin-up channel around the Fermi level at the ground state. This corresponds to the half-metallic transport phase. Thus, at zero bias, the bottom of the conduction band and the top of the valence band are dominated by contributions from the *d*-orbitals of Co irrespective of the nature of the tunnel barrier layer. However, when the electric field is switched on, beyond 0.1 a.u. the half-metallic electronic transport phase at zero field changes to a metallic phase (see Figure 3b,c). The critical point of this quantum phase transition (QPTs) is at 0.1 a.u. the spin-up of Co atoms became metallic from the insulator as shown in Figure 3a. Figure 3c shows the PDOS of the tunnel layer atoms at the ground state. The corresponding PDOS of the tunnel barrier region at finite fields are shown in Figure 3e,f. These suggest that B and N do not contribute a significant number of electronic DOS around the Fermi level. 

To understand the hybridization of orbitals at the interface, the PDOS of monolayer hBN is shown in Figure 3d–f. The transport character of the hBN barrier layer changes to metallic transport at finite fields from an insulating character at zero bias (see Figure 3d,e). Firstly, the p-orbital of N and B atoms dominate the DOS around the Fermi level. Deep in the conduction band at zero fields, p-orbitals of B atoms contribute a significant majority of electronic DOS. Secondly, the transport character of the stack remains metallic at a higher field of 0.3 a.u. However, the DOS shows that majority and minority spin density decreased between ~−2 and 1.9 eV as in Figure 3f. At nonzero applied fields the Fermi level DOS increases significantly up to 0.1 a.u, before saturating at 0.3 a.u. The electric field-induced DOS on the tunnel barrier atoms constitutes the main source of the spin magnetic moments localized on N, to induce the QPT seen in Figure 2a.

Figure 4 (top panels) shows the PDOS in Co(111)/MoS_2_/Co(111) heterostructure while the bottom panels show the effect of the applied electric field on the electronic structure of the monolayer MoS_2_ tunnel barrier. The electronic structure at different field strengths of 0.0, 0.1, and 0.3 a.u. is shown in Figure 4a–c. The degree of hybridization in the interface depends on the strength of the orbital overlap and inversely on the energy separation between them. The PDOS at different field strengths is dominated by the *d*-orbital of Co. The transport phase is characterized by the half-metallic electronic structure, in each case (see Figure 3a). The similarity of the PDOS in all field configurations implies that the electric field does not affect the electronic properties when the barrier region is MoS_2_. This could also be the effect of a simple superposition of the electronic structure of all materials in the stack [72].

The two tunnel barrier models respond differently to the applied electric field up to the same point in the applied field. Beyond this point the responses of the tunnel barrier layers are invariant. We show in the next section, that this is the quantum critical point of the electronic phase transition. In the hBN model, for instance, it is the N *p*-orbitals that dominate the Fermi level. By contrast, it is the *d*-orbital of Mo at 0.1 a.u. that dominates in the MoS_2_ model. In both cases, at 0.3 a.u. (Figure 3f and Figure 4f) the metallic phase is also dominated by the *p*-orbital of N and S atoms. However, the *p*-orbital of the S atom contributes far more states per eV around the Fermi level than the *d*-orbital of the Mo atom. Nevertheless, the main contribution to the MoS_2_ PDOS does not originate from the Mo atoms alone, as found in [73]. 

However, in the present work, we find that there is a competition between the *d* and *p*-orbitals of Mo and S atoms, especially at high electric field. When these trends are compared to the equal contribution of their orbitals at zero bias (see Figure 4d), and the disappearance of the native bandgap of the isolated MoS_2_ layer around the Fermi level in the electronic structure of the stack, as shown in Figure 4e,f, it becomes clear that the applied field plays an important role in coupling the component electronic structures in the stack and in the resulting topological behaviour in the spin transport. Also, the strong bonding between the MoS_2_ and Co (111) and the small distance between S and Co allow substantial wavefunction overlap between Co, Mo, and S states, like that found for Ti and MoS_2_/metal contacts [74,75]. Importantly, the PDOS becomes spin-polarized at the Fermi level, revealing the spin injection into MoS_2_.

Inspection of the PDOS shown in the top panels of Figure 3a and Figure 4a in both structures as well as the local PDOS of atoms within the insulating tunnel barrier (shown in the corresponding bottom panels) shows that the Fermi level of the spin down channel is dominated by Co 3*d*-states. This is created within the valence band by bonding hybrids between an admixture between the surface states of Co(111) and small electronic contributions from the 3*d*-states of Co and the 2*p*-states of both B and N atoms. At the Fermi level, the corresponding conduction band states are attributable to contributions from hybridization of the antibonding states of Co with electronic states that arise from short-ranged electrostatic disorder. Thus, the insulating gap in the up-spin channel is attributable to strong *p-d* hybridization between Co 3*d*-states and the 2*p*-states of both B and N atoms. The same arguments are valid for Mo and S atoms in the barrier. However, because of strong *d-d* coupling between the Co and Mo 3*d*-states, the electronic DOS maintains the wide insulating gap in the up-spin channel. Therefore, the gap in the up-spin channel is enforced by hybridization of the lower lying B and N states of the hBN-based system.

##### Variation of the Spin-Flip Energy with the Applied Electric Field 

**Figure 5:**
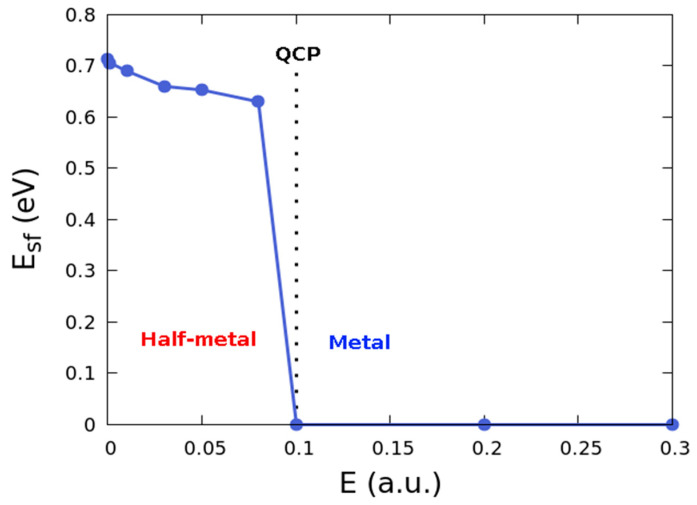
Carrier transport phase diagram of the Co(111)/hBN/Co(111) stack showing the quantum critical point (QCP) denoted by the dashed vertical line.

Figure 5 shows the spin carrier transport phase diagram in the Co(111)/hBN/Co(111) heterostructure. The phase diagram shows variations in a non-thermal control parameter denoted herein by the spin-flip energy ($E_{sf}$). This parameter is equivalent to the metric of local order in the Landau theory of phase transition since the QPT in the transport phase is driven by quantum rather than thermal fluctuations. The spin-flip energy has been calculated as the difference between the Fermi energy level and the highest occupied level [76]. Figure 5 shows a trivial phase transition at 0.1 Hartree a.u. at the quantum critical point (QCP) of the phase diagram. There is a decrease in the spin-flip energy as the electric field strength is increased to the QCP. Note that the spin-flip energy vanishes beyond the QCP. The QCP corresponds to the critical field at which the first order QPT occurs.

In the spin transport phase diagram shown in Figure 5, the local order parameter is the spin-flip energy. It correctly characterizes the macroscopic state of spin carrier transport in the device at 0 K. The predicted QPT is denoted by the sudden change from half-metallic to metallic character exclusively from the effects of the applied electric field. These demonstrate the efficacy of using electric fields to switch between distinct transport phases. It is crucial to provide a physically transparent justification that the mechanisms involved in the change from half-metal to full metal transport can be considered as a quantum phase transition (QPT). To qualify as a QPT, the fluctuations must be driven by quantum fluctuations at 0 K [3,4,5]. Since the number of degrees of freedom to consider is very large in a macroscopic many-body solid, the quantum fluctuations cannot be attributed to changes in the average number of atoms *N* in the system. This is because average fluctuations in the number density vanishes in the thermodynamic limit in a macroscopic many-body solid. Instead, it is the quantum fluctuations in the electronic structure that bears all the information required for triggering the QPT. 

To understand how the observed QPT is driven by quantum fluctuations at 0 K, we note that fluctuations of the zero-field ground state are not spontaneous. Since the observed magnetization (Table 1) is dependent on the electric field, its saturation cannot be attributed to collective spin ferromagnetism. We ascribe it to the giant orbital paramagnetism associated with the collective response of electrons in coherent domains due to the associated Berry connection [77]. This interpretation is based on the model for mesoscopic structure formation when zero-point fluctuations of the vacuum electromagnetic field interact with an ensemble of two-level atoms, at 0 K [78]. In addition, consider that the SOC plays no role in breaking time reversal symmetry. The quantum fluctuations are driven by the applied potential bias due to spontaneous breaking of time-reversal symmetry in the spin space. This occurs only when the external electric field is applied. Thus, the magnetic ground state is favored at 0K over the fully spin-paired nonmagnetic state by lowering the ground state energy. This mechanism only occurs by creating an imbalance in the spin carrier population between the up and down spin states around the Fermi level on some atomic sites. 

Figure 2 shows the effect of the resulting quantum fluctuation in carrier population on the magnetic sites of Co. The Co atoms of different layers clearly manifest a fluctuation of the localized moments as applied field changes relative to the zero-field moments. The field induced quantum fluctuations also manifest as an imbalance in moments localized on intrinsically nonmagnetic ions, such as N, B, Mo, and S. The non-spontaneity of the quantum fluctuation in our DFT calculations is understandable from the perspective of the vanishing partition function at ground state of a many-body solid. Yet, as the external field is introduced to the system at 0 K, the new ground state is no-longer necessarily a microstate of the zero-field Hamiltonian. In both extremal limits of the applied field, it is the changes observed in the spin carrier transport phase that encapsulates the unique signatures of the underlying quantum fluctuations. The conventional thermodynamic interpretation of a first order phase transition involves either a gain (or loss) of latent heat. During such phase transitions, the physical system either absorbs or releases a large but constant amount of energy per unit volume. Although similar principles are valid in our case, the first order phase transition anticipated herein is exclusively an electronic phase transition. 

Table 1 shows that a finite amount of energy is lost by the electronic system at the QCP. This manifests as a relatively more negative total energy at 0.1 a.u. This observation is ascribed to the field induced quantum fluctuations at 0 K. Domination of the quantum fluctuations result in decreased total energy. The effect is an increased occupancy of states around the Fermi level, and this causes the observed crossover of the phase boundary from one carrier transport phase to another (e.g., from half-metal to metal phase). This phase crossing ultimately decides the spin transport behavior, or the anisotropy exhibited by the pMTJ in an all-spin logic device. A closely related phenomenon is also obtainable when the quantum phase transition is topological rather than trivial.

Under strong SOC, the effect of applying a perpendicular external magnetic field to the heterostructure leads to the emergence of robust magnetic skyrmion spin textures in artificially stacked multilayers [70]. We have shown recently in Ref. [79] that it is facile to switch the electronic transport to topologically ordered quantum phases from such a trivial ground state when the bulk electronic structure is renormalized to a 2D model of carrier transport via a manifold of the low-energy fully gapped ground state on the honeycomb lattice. Consider that the Coulombic interaction between the two isolated ferromagnetic leads and the monolayer tunnel barrier layer is a symmetry-breaking first-order perturbation of the electronic structure leading to proximity effects [80,81], since the heterostructure structure is electrostatically coupled. Thus, external bias fields such as gate potential, external electric, and magnetic field in pMTJs [82,83,84] is likely to create the magnetoelectric couplings observed herein to mediate the emergence of exotic collective phenomena. Some of such phenomena include spin-polarized charge density wave phase [21], the emergence of robust magnetic skyrmion spin textures [70], and formation of a quantum-fluid phase at distinct quantum phase transition points [79]. In Section 3.3, we analyse the observables of the electronic structure that uniquely characterize the proximity-induced magnetoelectric coupling to clarify the nature of the observed QPT. 

### 3.3. Magnetic Proximity Effect

The magnetoelectronic couplings reported hereunder are nonlinear effects of the electric field. These are observed as electric polarizations induced by the spatial modulation of the spin current density. These responses characterize the behavior of the spin current produced in the perpendicular magnetic tunnel junction when it is subjected to varying magnitude of the applied electric field. 

#### 3.3.1. Interfacial Charge Transfer 

**Figure 6:**
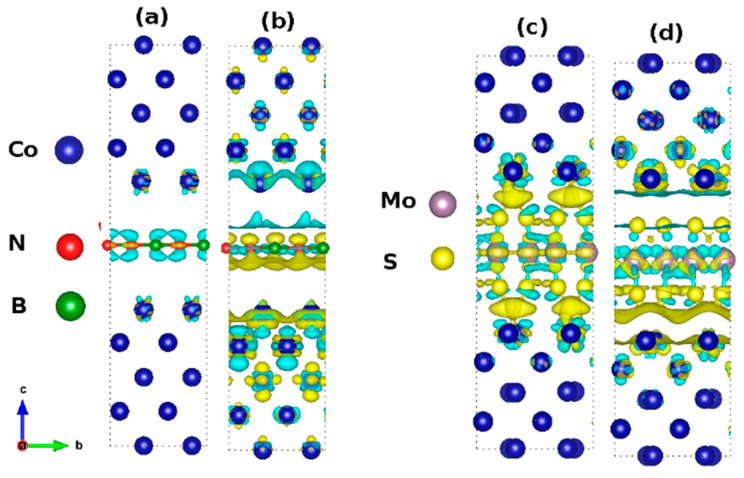
Difference charge density for a stack containing monolayer hBN (left panel) and MoS_2_ (right panel) tunnel barrier at applied electric field of amplitude 0.0 [(**a**,**c**)] and 0.1 a.u. [(**b**,**d**)]. Yellow and cyan denote electron accumulation and depletion regions, respectively.

Figure 6 show the 3D charge density difference ∆ρ(r) map under the influence of the applied electric field. This illustrates the charge transfer in the heterostructure, as a function of the applied electric field. In each case, the yellow and cyan colors correspond to a local excess and a local depletion of electrons. The charge distribution at the interface provides an intuitive understanding of the interactions between Co(111) and the tunnel barrier discussed herein. Figure 6 shows the ∆ρ(r) in Co(111)/hBN/Co(111) stack plotted at an isosurface value of 1 × 10^−3^ e/Å^3^. A net transfer of charges has occurred in the interface between the Co and N atoms at 0.0 (Figure 6a) and at 0.1 a.u. (Figure 6b). A high charge accumulation is also observed on the N atoms. This transforms the hBN monolayer to an electron-rich layer, while electron depletion at the Co makes it a hole-rich region (Figure 6b). The charge distribution around the Co/hBN interface shows a large swath of smoothly connected regions in both upper and lower interfaces where the electron density vanishes. 

Figure 6c,d show the ∆ρ(r) for the Co(111)/MoS_2_/Co(111) heterostructure at an isosurface value of 3 × 10^−3^ e/Å^3^. There is a strong overlap of electron density between Co and S atoms at the interface. The transfer of charges along the covalent Co-S bonds leads to strong structural integration. Figure 6c shows a strong accumulation of charges on the Co-S bonds when no electric field is applied. The charge accumulation regions manifest as an electronic cloud formed at the tunnel barrier. This is shown in yellow at the interface and suggested this region is full of electrons. As the magnitude of the applied field is increased to 0.1 a.u., the top interface becomes an electron-deficient region or holes rich due to the charge depletion. The accumulated charges form a localized region of flat shape in the top interface and the equivalent region in the bottom interface has a wavy shape, as shown in Figure 6d.

**Figure 7:**
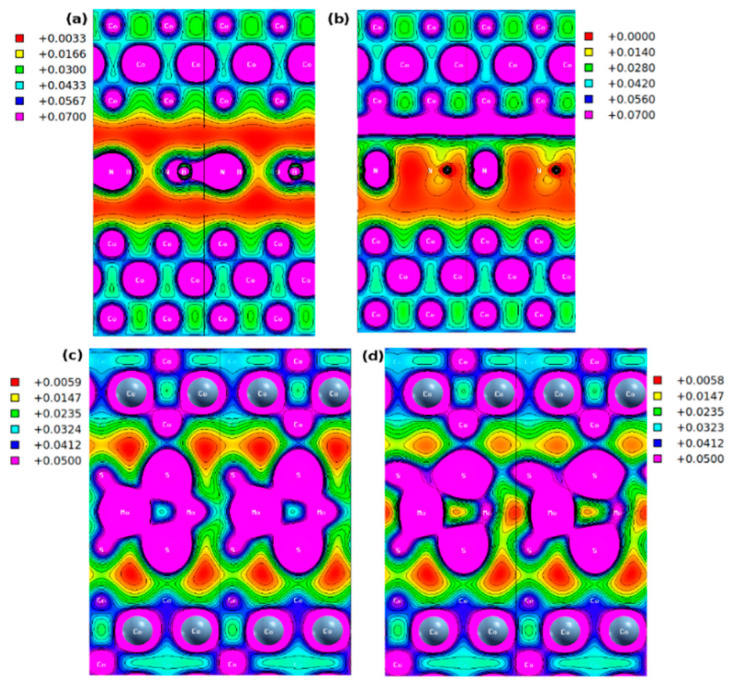
Contour plots of the total (volumetric) charge density distribution in the [110] plane at 0.0 (**a**) and 0.1 a.u. (**b**) electric field. The top panels denote Co(111)/hBN/Co(111) while the bottom panels denote Co(111)/MoS_2_/Co(111) at 0.0 a.u. (**c**) and 0.1 a.u. (**d**).

Figure 7 shows the 2D projection of the total (volumetric) charge density distribution in the [110] plane. The distribution of the volume charge density in the region between Co(111) and hBN is 3.3×10^−3^ e/Å^3^ within the Co(111)/hBN/Co(111) stack (Figure 7a). This magnitude of the charge density is low and represents only 4% of the maximum charge density localized on the B-N bond in the interface. This is similar to the low charge density region localized between Fe atoms of the Fe(110) layer that makes interfaces with the hBN layer in the more complex graphene/hBN heterobilayer barrier [23]. Similarly, Figure 7b shows the volume charge density distribution in Co(111)/hBN/Co(111) heterostructure for an applied electric field of 0.1 a.u. This shows that the region of the maximum and minimum charge density localization is switched. This is a unique signature of the proximity effect in the symmetric tunnel junction. 

A closer look at Figure 6b reveals a contiguous line of zero charge density region surrounding the puddles of localized high charge density with the hBN monolayer tunnel barrier inserted. This suggests that carrier transport occurs through site hopping in the half-metallic phase. The applied electric field, which breaks the symmetry of the charge distribution at the ground state, introduces a contiguous region of high carrier density only on one side of the interface. This nanoroad leads to full metallic transport. Here, the spin nanoroad denotes the unique 1D-like conducting channel that manifests in the spin-resolved volume charge density of the metal-insulator-metal heterostructure permitting the flow of spin-polarized carriers in a hybrid material. Jung, et al. [85] introduced the “nanoroad” concept into the analysis of the carrier transport properties of graphene conducting channels that were incorporated into the insulating boron nitride sheets. 

A similar nanoline transport channel for spin carriers is not observed with the MoS_2_ tunnel barrier. Therefore, we conclude that the applied electric field is acting like a toggling switch of the charge density. Figure 7a,b also show that there are sites of maximum charge density localized on ionic cores. These suggest the existence of a dipole across the interface due to the applied electric field. Figure 7c,d show the corresponding total charge density with the MoS_2_ tunnel barrier There are non-contiguous pockets of low charge density regions within the interface. Crucially, the electric field has minimal effect on the charge density relative to the ground state distribution. 

Consider that the formation of the observed spin nanoroad is an induced effect of the electric field that is observable only when the monolayer hBN is integrated into the stack. This effect is only observed when the magnitude of the applied field is 0.1 a.u. We have ascribed the formation of the spin nanoroad conducting channel to the consequence of the spin crowding phenomenon. Firstly, this interpretation is physically motivated by our findings that the nanoroad is not observed at lower electric fields in our calculation. Secondly, spin crowding denotes the microscopic processes that culminate in the formation of the nanoroad from self-diffusion of spin-polarized volume charge density domains. In this case, the build-up of localized spin density regions and their migration to form a contiguous spatial region culminates in the spin current crowding effect. This is due to the transfer of angular momentum of carriers from electrons to the ions that make up the stack, as they move under the effect of the applied electric field. 

The current crowding phenomenon is well-known in nanostructured semiconductors wherein the charge density is distributed non-homogenously. For instance, recently, it has been observed in the current density distribution profile around the metal insulator transition point in NdNiNO_3_ thin film with a thickness gradient [86], and in interface engineered electrical contacts [87]. Current crowding is also observed in superconducting nanostructures [88], wherein the contiguous current density region is considered a superconducting condensate. In each of the cases, the contiguous charge density regions are analogous to the spin current density region by the spin nanoroad observed in our study. 

#### 3.3.2. Barrier-Dependence of Spin Conductance 

Table 3 and Table 4 show electric field effects on parallel (P) and anti-parallel (AP) spin conductance in Co(111)/hBN/Co(111) and Co(111)/MoS_2_/Co(111), respectively. Herein, $G^{↑↑}$ and $G^{↑↓}$ denotes the spin conductance in P and AP alignments while the transmission coefficients for the majority ($T^{↑}$) and minority ($T^{↓}$) spins are shown in units of $e^{2}/h\;$ = 3.874 × 10^−5^ $\Omega^{-1}$. Table 3 shows the spin asymmetry with the hBN tunnel barrier. The spin conductance of the P-alignment is higher than the conductance of the AP-alignment at all amplitudes of the applied electric field. This suggests that there is a large resistance to spin transmission in the AP configuration. We have attributed this resistance to dissipative spin scattering due to chiral anomalies in the Fermi level topology of the up and down spin fields [21]. This resistance causes the observed differences in spin conductance between the P and AP alignments as the magnitude of the electric field changes. For instance, the spin conductance is 0.0001 and 0.00004 $\Omega^{-1}$ in the P and AP configurations, respectively. This is due to the higher transmission of spin carriers in the P configuration. However, the conductance of the AP alignment is higher at 0.3 a.u. than the conductance of the P alignment.

Table 4 shows a comparatively lower spin conductance with the MoS_2_ tunnel barrier. The same trend is also seen for conductance variations with the applied field. It is also important to note that even though the spin transmission coefficients for the majority ($T^{↑}$) and minority ($T^{↓}$) spins, and the corresponding spin conductance $G^{↑↑}$ and $G^{↑↓}$ are not measurable, they are closely related to the spin polarization P. In the hBN-based stack, for instance, we have used 2.0 $e^{2}/h$ for the spin-up transmission ($T^{↑}$) and 0.74 $e^{2}/h$ for the spin-down transmission ($T^{↓}$) to obtain a spin polarization of 46% in the P configuration using *η* = $\frac{T^{↑}\left(E_{F}\right)-T^{↓}\left(E_{F}\right)\;}{T^{↓}\left(E_{F}\right)+T^{↑}\left(E_{F}\right)\;}$. Similarly, the calculated spin polarization is 57% in the AP configuration. Because of the electric field, the spin polarization increases to 99% in the P configuration but reduces to 11% in the AP configuration at 0.3 a.u.

By contrast, the spin transmission is high in the MoS_2_ based stack when no external field is applied. The transport character is metallic, and transport is dominated by minority spin channel ($T^{↓}$) for P and AP configurations. The metallic band structure at the ground state is invariant under the applied electric field. Conductance in the P configuration is dominated by the majority spin while the conductance in the AP configuration is dominated by the minority spin. Using 1.32 $e^{2}/h$ and 1.18 $e^{2}/h$ for the majority and minority spin channels in the P configuration respectively, a small polarization of 5% is obtained in the P configuration while 62% is obtained in the AP configuration. At 0.3 Hartree a.u, the P configuration polarization is 82%. In the AP configuration, the transmission coefficient is 0.005 ($T^{↑}$) and 0.003 ($T^{↓}$). These lead to identical spin polarization of 19% in both channels. Thus, a straightforward and intuitive way to reach high spin polarization of the current with pMTJs is to incorporate half-metallic electrodes. 

**Figure 8:**
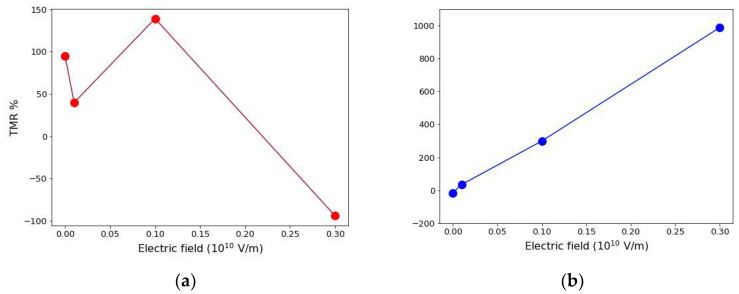
Dependence of the tunneling magnetoresistance on the tunnel barrier material for different magnitudes of the applied electric field for hBN (**a**) and MoS_2_ (**b**).

Figure 8 shows the effect of the applied electric field strength on the TMR for the two tunnel barriers. The TMR is 94.95% with hBN at zero applied field (Figure 8a). The applied electric field causes a significant fluctuation in the calculated TMR. The highest value of the TMR is 139.06%. This is obtained at an applied field of 0.1 a.u. The high TMR is due to the large spin transmission coefficient obtained in the majority channel of the P magnetic configuration. Since the TMR is positive when the two leads are made of the same material [9], the observed decrease in TMR to −94% at 0.3 a.u. (see Figure 8a) is an effect of magnetoelectric coupling. Negative TMR at higher applied fields corresponds to the strong asymmetric bias dependence. This behavior is due to both the energy dependence of the spin polarized PDOS and the type of tunnel barrier used. Bonding mechanisms at the Co(111)/tunnel barrier interface control the efficiency of interfacial spin transmission. Crucially, the amplitude at which the applied electric field causes the QPT in the presence of the hBN tunnel barrier corresponds to the amplitude at which the TMR reaches its maximum value. Beyond this saturation point, the TMR decreases continuously. 

Figure 8b shows that the TMR increases monotonically to 299.09% at 0.1 Hartree a.u. and a maximum value of 987.02% at 0.3 a.u. when the stacks contain the MoS_2_ tunnel barrier. The above trend agrees well with the colossal magnetoresistance of 300% in the Fe/MoS_2_/Fe pMTJs reported in ab initio transport calculations by Dolui et al. [73]. The monotonic increase in the TMR with MoS_2_ is attributable to a more efficient spin filtering effect in the AP spin configuration. The origin of the negative TMR observed in Figure 8a is attributable to the following underpinning mechanisms. Firstly, the observed quantum conductance is larger in the AP alignment of spin than in the P alignment (see Table 3 and Table 4). Secondly, the blocking of spin transport through the minority channel of hBN especially in the AP configuration is due to the high resistance induced by dissipative scattering from chiral anomalies in the Fermi level topology. 

In the regime of small (but non-vanishing) SOC and broken inversion symmetry, which is applicable in the structures considered herein, the spin blocking proceeds through the Dyakonov-Perel mechanism. In metallic systems with inversion symmetry, the above mechanism corresponds to the Elliot-Yafet mechanism, and any scattering event that yields no spin-flip causes the admixing of pure spin states leading to spin relaxation [89]. Overall, the estimated magnetoconductance of the pMTJ is denoted by the TMR obtained with the two tunnel barriers (see Figure 8a,b). This agrees well with those reported in the literature for symmetric pMTJs with MoS_2_ and hBN barriers [90]. We conclude that using either hBN or MoS_2_ as the tunnel barrier layer exhibits excellent spintronic performance because the calculated TMR: 139% (hBN) and 987% (MoS_2_) show significant improvements compared with the TMR obtained with the same Co electrodes using Al_2_O_3_ tunnel barrier [91,92].

**Figure 9:**
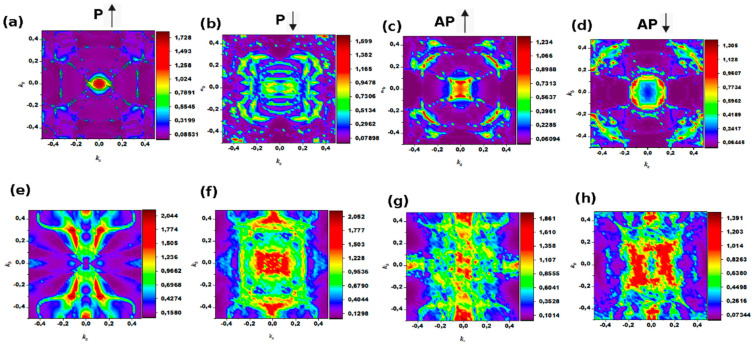
**k_||_**-resolved spin transmission spectra showing the majority (i.e., up) and minority (i.e., down) spin states, respectively, at the critical field of 0.1 Hartree a.u. in pMTJs based on monolayer hBN barrier in parallel (P) configuration (**a**,**b**) and antiparallel (AP) configuration (**c**,**d**), and in pMTJs based on monolayer MoS_2_ in parallel configuration (**e**,**f**) and antiparallel configuration (**g**,**h**).

To gain a deeper understanding of the relationship between hybridization of orbitals and the magnetoelectric couplings, we have also calculated the *k*-space-resolved spin transmission coefficients as a projection onto the 2D Brillouin zone (BZ) of the interface at the critical field. Figure 9 shows the wavevector dependence of the up and down spin transmission coefficients in the P and AP configurations as a contour plot. Figure 9a indicates that the majority spin channel of the Co/hBN/Co multilayer (top panel) in P and AP configurations dominates the spin transmission relative to the minority spin. Moreover, the dense hot spots originate from the center of the BZ (Γ-point), as indicated by the color scale. This confirms that spin-up electrons have higher transportability than spin-down electrons. For the hBN-based model, the positive transmission of majority spin at the Γ-point of the BZ in the P configuration arises from the strong coupling between occupied and unoccupied spin-down states of the Co *d*-orbitals. This is also in good agreement with transmission calculations (see Table 3 and Table 4).

In the AP configuration, the spin transmission corresponding to the majority and minority spins are strong and weak around the Γ-point, respectively. This conclusion is also in good agreement with our layer-resolved spin-polarized charge density values for the Co atoms in the hBN/Co interfaces in Figure 7b. The hBN-based pMTJ shows minimal contributions from carriers at the Brillion zone edges and corners in the AP configuration. One can also see in the AP configurations shown in Figure 9c,d that the hot spots region originates from Γ-point in spin-up. However, the minority spin for P configuration is weakly coupled to the wave vectors, and this is characterized by low density (see Figure 9b). For the AP configuration, the low density at the Γ-point is surrounded by hotspots at the corner.

On the other hand, for Co/MoS_2_/Co heterostructure (bottom panel), there is an opposite situation that in P. The minority spin channel has a much larger transmission than the majority channel (Figure 9e,f), and Γ-point dominates the highest spin density. However, in an AP configuration, the down spin transmission is characterized by the re-appearance of hot spots around the Γ-point, and the transmission is more pronounced at the edges of the Brillouin zone and with minimal contributions through the zone corners. Overall, the transmission in both cases is reasonably large, which opens the possibility for spin injection into MoS_2_ and hBN and confirms that the Co electrode is an efficient spin injector for the device.

## 4. Conclusions

To summarize, we have presented an ab initio study of the magnetoelectric coupling and proximity effects that may arise from the effect of a perpendicular electric field on the spin transport in symmetric Co(111)/hBN/Co(111) and Co(111)/MoS_2_/Co(111) hybrid multilayers. Our results show that the electronic structure of the Co(111)/hBN/Co(111) pMTJ undergoes an electric field-induced quantum phase transition from the half-metallic to metallic phase due to a strong hybridization of *p*-orbitals of N and B atoms with *d*-orbital of Co at the interface. Nevertheless, the electronic structure in the MoS_2_ model remains unaffected by the electric field. Analysis of the field-dependent charge transfer shows a contiguous line of high charge density on one side of the Co(111)/hBN interface. This shows the formation of a spin nanoroad due to electric field-induced spin crowding. Our calculations reveal a giant tunnelling magnetoresistance of 987.02% and 139.06% in MoS_2_ and hBN models, respectively. The magnitude of the applied electric field at the quantum critical point corresponds to the electric field at which the tunnelling magnetoresistance saturates in the presence of a monolayer hBN tunnel barrier. These offer insights into the origins of dissipative scattering anomalies in all-spin logic devices, as a crucial first step toward a lower energy-delay performance and faster switching operations in spintronics.

## Figures and Tables

**Table 1:** Field effects on the magnetization energy (Δ*E*), magnetization (*M*) per cell (in Bohr magneton) for spin aligned parallel (P), and antiparallel (AP) to the quantization axis.

Electric Field		Co(111)/hBN/Co(111)			Co(111)/MoS_2_/Co(111)		
a.u.	10^10^ V/m	*M* (P)	*M* (AP)	Δ*E* (eV)	*M* (P)	*M* (AP)	Δ*E* (eV)
0.0	0.0	62.6	−0.03	0.0	62.34	−0.57	0.0
0.01	0.51	62.5	0.32	−0.004	62.49	−0.48	0.002
0.1	5.14	18.3	4.31	−2.59	62.33	−0.28	0.038
0.3	15.43	28.0	12.25	−1.49	61.14	61.13	−1.95

**Table 2:** Sensitivity of the spin order and magnetic exchange coupling to the tunnel barrier material and the applied electric field.

Electric Field		Co(111)/hBN/Co(111)		Co(111)/MoS_2_/Co(111)	
a.u.	10^10^ V/m	Spin Order	*J* (meV)	Spin Order	*J* (meV)
0.0	0.0	Half-metallic	0.0139	Half-metallic	0.0181
0.01	0.51	Half-metallic	0.0132	Half-metallic	0.0184
0.1	5.14	Metallic	−0.1211	Half-metallic	0.0188
0.3	15.43	Metallic	−6.1618 × 10^−4^	Half-metallic	7.7579 × 10^−7^

**Table 3:** Effect of electric field on the spin conductance of the Co(111)/hBN/Co(111) stack.

**Electric (a.u.)**	Field (10^10^ V/m)	Parallel			Anti-Parallel		
		$T{\;}^{↑}$	$T^{↓}$	$G^{↑↑}$	$T^{↑}$	$T^{↓}$	$G^{↑↓}$
0.0	0.0	0.27284	0.93178	4.66	0.3192	0.2987	2.394
0.01	0.51	0.25774	0.71976	3.79	0.3944	0.3053	2.71
0.1	5.14	2.014	0.74109	100	0.8513	0.2284	4.183
0.3	15.43	0.03491	0.0000063	0.135	0.3801	0.2963	2.621

**Table 4:** Effect of electric field on spin conductance of Co(111)/MoS_2_/Co(111) stack.

**Electric (a.u.)**	Field 10^10^ (V/m)	Parallel			Anti-Parallel		
		$T^{↑}$	$T^{↓}$	$G^{↑↑}$	$T^{↑}$	$T^{↓}$	$G^{↑↓}$
0.0	0.0	0.9103	0.1087 × 10^1^	7.730 × 10^−5^	0.688	0.165 × 10^1^	9.08 × 10^−5^
0.01	0.51	0.1321 × 10^1^	0.118 × 10^1^	9.695 × 10^−5^	0.4356	0.1417 × 10^1^	7.17 × 10^−5^
0.1	5.14	0.1795	0.182 × 10^1^	7.748 × 10^−5^	0.24	0.2611	1.94 × 10^−5^
0.3	15.43	0.849 × 10^−1^	0.853 × 10^−2^	3.622 × 10^−6^	0.51 × 10^−2^	0.346 × 10^−2^	3.33 × 10^−7^

## Data Availability

The data presented in this study are available in the articles.
